# Trend of pulmonary tuberculosis and rifampicin-resistance among tuberculosis presumptive patients in Central Tigray, Ethiopia; 2018 -2023: a six-year retrospective study

**DOI:** 10.1186/s40794-024-00224-1

**Published:** 2024-07-01

**Authors:** Guesh Gebremariam, Mulugeta Kiros, Selemun Hagos, Haftom Hadush, Amaha Gebremichael, Gebretsadkan Gebrekirstos, Aregawi Tesfay, Teumelsan Gebrewahid, Tesfay Berihu, Brhane Gebremariam

**Affiliations:** 1 Department of Medical Laboratory Science, Unit of Medical Microbiology, College of Health Science, Aksum University, Axum, Ethiopia; 2 Department of Medical Laboratory Science, Unit of Medical Parasitology, College of Health Science, Aksum University, Axum, Ethiopia; 3 Department of Medical Laboratory Science, Unit of Clinical Chemistry, College of Health Science, Aksum University, Axum, Ethiopia; 4 Department of Biomedical Science, Unit of Anatomy, College of Health Science, Aksum University, Axum, Ethiopia; 5 Department of Medical Laboratory Science, Unit of Medical Microbiology, College of Health Science, Raya University, Maichew, Ethiopia

**Keywords:** Trend, Tuberculosis, Retrospective, X-pert MTB/Rif assay, Central Tigray

## Abstract

**Background:**

Tuberculosis (TB) is a major public health concern in the developing countries. Moreover, the emergence of multidrug-resistant tuberculosis is challenging. However, there are no organized data on the trends of pulmonary tuberculosis and rifampicin-resistant *Mycobacterium tuberculosis* in the study area.

**Methods:**

A retrospective cross-sectional study was conducted to fill the information gap in Central Tigray at St. Mary General Hospital between 2018 and 2023. Data were collected from the GeneXpert™ tuberculosis registration logbooks using standard checklists and analyzed using Statistical Package for Social Science version 22. After performing logistic regression, a p-value < 0.05 with a corresponding 95% confidence interval was considered statistically significant. Moreover, chi square test for trend was performed to assess the percentage of annual detection of pulmonary tuberculosis and rifampicin-resistant *Mycobacterium tuberculosis* during the study years.

**Result:**

Presumptive pulmonary tuberculosis patients with complete data (*n* = 3696) were included in the study. The overall prevalence of pulmonary tuberculosis was 11.7%, of which 8.1% were resistant to rifampicin. The study revealed that the incidence of pulmonary tuberculosis has been increasing, mainly in the recent four years. Likewise, an increase in rifampicin-resistant *Mycobacterium tuberculosis *was observed with considerable fluctuations. Age, human immunodeficiency virus infection, and presumptive rifampicin-resistant *Mycobacterium tuberculosis *infection were significantly associated with the presence of pulmonary tuberculosis. Moreover, pulmonary tuberculosis was more prevalent among participants in the productive-age group.

**Conclusion:**

Although there have been fluctuations, an increasing of pulmonary tuberculosis and rifampicin-resistant *Mycobacterium tuberculosis* has been observed in recent years. Hence, prevention and treatment strategies for tuberculosis should be strengthened to alleviate the burden of pulmonary tuberculosis and rifampicin-resistant *Mycobacterium tuberculosis *in the study area.

## Introduction

Tuberculosis (TB) is an infectious disease mainly caused by *Mycobacterium tuberculosis* (MTB), which is transmitted from infected individuals to healthy individuals via aerosol transmission [1]. MTB bacteria affect all age groups equally; however, TB develops in only 10% of humans who are exposed to it, whereas the remaining 90% develop tuberculosis infection (TBI).Active TB has a higher dose of TB bacilli than TBI, and acts as an infection source for contacts [2]. TB are two forms; pulmonary tuberculosis (PTB) and extrapulmonary tuberculosis (EPTB) [3]. PTB is the most widely recognized form of TB, accounting for 80% of all TB cases globally [4]. TB is a curable and preventable disease. However, if not effectively treated, the disease is lethal [5].

Although progress is being made to reduce TB globally, it remains to be the second-leading infectious killer followed by Covid19 with 10.6 million TB cases and 1.4 million deaths in 2021 [6, 7]. Regardless of its global distribution, TB excessively affects people in resource-poor settings, particularly in Asia and Africa. More than 90% of new TB cases and deaths occur in the developing countries [7]. The dramatic growth of TB in developing countries is triggered mainly by the emergence of Human Immunodeficiency Virus (HIV) [8].

Ethiopia was included among 30 high TB burdened countries in the globe [9]. Despite the positive progress of TB management to meet the “END TB Strategic goal” of 2030 that is aimed at reducing the death rate by 90%, TB in Ethiopia is still the third cause of hospital admission and the second highest cause of mortality [10, 11]. According to the World Health Organization (WHO) global TB report, 117,705 cases and 28,600 deaths due to TB have been reported in the country [12]. Poor socioeconomic status, lack of healthcare service utilization and access, delays in seeking care and diagnosis, and poor knowledge about the disease are among the contributors to active TB case notifications [13].

Despite the availability of highly effective treatment [1], the emergence and spread of multidrug-resistant tuberculosis (MDR-TB) pose a threat to human health worldwide. This complicates the diagnosis, treatment, and control of TB diseases [14]. Drug-resistant TB also plays a valuable role in increasing the burden of communicable and non-communicable diseases [11]. Thus, rapid detection, early treatment initiation, continuous surveillance, and regular monitoring of drug-resistant TB are essential for disease management and control programs [11, 15]. Developing countries are more highly affected by drug-resistant TB than developed countries because of different factors, including inadequate resources for early detection, high prevalence of HIV, inappropriate TB treatment, and late detection of TB cases [11, 16, 17]. In 2020, the World Health Organization (WHO) global TB report indicated that there were approximately 206,030 new cases of Multi drug resistant tuberculosis (MDR-TB), representing a 10% increase from 186,883 in 2018 [18, 19]. Despite using directly observed therapies (DOTs) that encourage treatment success [6], more than 5800 estimated MDR-TB cases are emerging in Ethiopia yearly [9]. The magnitude of the RR-TB in these countries ranged from 3.5% to 43.5% [7, 20]. Therefore, determining the trend of PTB and detecting the rate of RR-TB is warranted to improve the performance of DOTs services, active TB case notifications, and devise a national TB management plan.

Although a few studies [7, 11, 21] have been conducted in other parts of Ethiopia, the magnitudes of PTB and MDR/RR-TB varied from place to place, and there were no documented studies regarding the trend analysis of PTB and MDR/RR-TB in the study area. Hence, this study aimed to evaluate trends in PTB and MDR/RR-TB from 2018 to 2023 in Central Tigray, Ethiopia.

## Methods

### Study design, area and period

This hospital-based retrospective cross-sectional study was conducted at St. Mary General Hospital between 2018 and 2023. The axis represents the zonal administrative city of central Tigray, which is found 1042 km from the capital city of Ethiopia, Addis Ababa. It is located at a latitude of 14°6’N and longitude of 38°17′E, at an elevation of 1953 m (4017 feet) above sea level. According to the Ethiopian Statistics Service, Axum has a total population of 94,515 (45,924, males and 48,591 females) [22]. In Central Tigray, there are 6 primary hospitals, 61 health centers, 3 general hospitals, and 1 referral hospital [23]. St. Mary General Hospital is a general hospital in the central zone of Tigray that provides medical services to more than two million people [24].

### Eligibility criteria and study variables

Those who had complete data in the GeneXpert TB registration logbooks during the specified study period were included, and cases with intermediate/invalid data were excluded from the study. PTB and RR-TB were dependent variables. Furthermore, sex, age, co-infection, presumptive diagnosis of PTB/MDR-TB, residence, and year of diagnosis were independent variables in this study.

### Study population

All TB presumptive clients with complete laboratory data in the GeneXpert TB registration logbooks comprised the study population.

### Laboratory diagnosis of PTB

The St. Marry General Hospital TB Clinic operates under the national TB and leprosy control program in Ethiopia. Samples of presumptive TB patients were diagnosed using both the GeneXpert MTB/RIF assay according to the manufacturer’s manual and microscopic examination according to the standard operating procedure (SOP). However, we preferred to include the results of the GeneXpert MTB/RIF assay because of its increased sensitivity and ability to detect RR-TB. All experimental protocols were approved by an institutional laboratory technologist.

### Data collection and quality assurance

Data were collected from the GeneXpert TB Laboratory Registration Logbooks at St. Mary General Hospital using a standard data collection format adopted from the WHO checklists by trained data collectors under the supervision of the principal investigator. The checklists contained unique codes (patient’s medical record number to be confidential for the patient results), age and age categories (using the formula: K = 1 + 3.322 log (*n* = 3696), sex, residence (urban/rural), HIV status (positive/negative/unknown), year of diagnosis, clinical diagnosis (PTB/MDR/RR-TB), and results of the GeneXpert MTB/RIF assay (PTB not detected; PTB detected, RR not detected; PTB detected, RR detected). Data clarity and completeness were checked daily by a principal investigator.

### Data processing and analysis

The collected data were cleaned and edited using EPI Info version 7, checked for completeness, clarity, and consistency, and exported to the Statistical Package for Social Sciences (SPSS) version 25 for analysis. Descriptive statistics for the different variables were calculated and presented in the form of text, tables, and graphs. Percentages, means, and standard deviations were used to generalize the results. Multicollinearity of independent variables was checked using variance inflation factor (VIF) and none was found. Binary and multivariable logistic regression analyses were used to examine the association between the dependent and independent variables. Variables with *p* < 0.25 in the binary logistic regression, were entered into multivariable logistic regression analysis to compute the adjusted odds ratio (AOR) to ascertain the degree of association between the risk factors of TB. In the multivariable logistic regression, a p-value less than 0.05, with a corresponding 95% confidence interval (CI), was considered statistically significant. Assumption on fitness of goodness of the model was checked by Hosmer and Lemeshow test and was found fit. Moreover, chi square test for trend was performed to assess associations (linear by linear association) of proportion of PTB and RR-TB detection by the year of report at 5% critical value.

### Operational definition

#### Presumptive pulmonary TB

An individual who presents with symptoms or signs suggestive of pulmonary tuberculosis as sweating, coughing for more than two weeks, loss of appetite, weight loss, and weakness.

#### Presumptive multidrug-resistant tuberculosis MDR-TB

Patients with TB relapse, lost to follow-up, and in close contact with persons infected with confirmed drug-resistant TB.

#### MDR-TB

MTB does not respond to isoniazid and rifampicin, the most important first-line anti-TB drugs.

#### RR-TB

MTB resistance to rifampicin detected using genotypic or phenotypic methods, with or without resistance to other first-line anti-TB drugs.

## Results

### Socio-demographic and clinical characteristics of study participants

A total of 4536 presumptive PTB patients who provided sputum samples were examined for PTB using the Gene Xpert MTB/RIF assay. Of those, 3696 (81.5%) completed the data and were included in the study. Among them, 59% and 87.9% of the study participants were male and PTB presumptive, respectively. Three-fourths, 2942 (79.6%) of the participants were aged 28 years and above, and the mean age of the participants was 46 (± 19 SD) with an age range from 1 to 97 years. Similarly, more than half, 1940 (52.5%) of the participants were screened for HIV during the TB investigative period, and 60.9% of the participants had urban residences. Furthermore, 3247 (87.9%) participants were presumptive for PTB (Table 1).

**Table 1:** Distribution of PTB with socio-demographic and clinical characteristics among presumptive patients in Central Tigray, Ethiopia from 2018 to 2023 (*n* = 3696)

Variables	Frequency, N (%)	Detection rate of PTB, N (%)
**Sex:**		
Male	2203[59.6]	254[11.5]
Female	1493[40.4]	180[12.1]
**Age:**		
≤ 13	156[4.2]	20[12.8]
14–27	506[13.7]	77[15.2]
28–41	913[24.7]	148[16.2]
42–55	893[24.2]	91[10.2]
56–69	745[20.2]	63[8.5]
70–83	437[11.8]	30[6.9]
84–97	46[1.2]	5[10.9]
**HIV Status:**		
Positive	86[2.3]	35[40.7]
Negative	1854[50.2]	284[15.3]
Unknown	1756[47.5]	115[6.5]
**Presumptive:**		
PTB	3247[87.9]	336[10.3];
MDR/RR-TB	449[12.1]	98[21.8];
**Residence:**		
Urban	2250[60.9]	198[8.8];
Rural	1446[30.1]	236[16.3];
**Years:**		
2018	312[83.6]	61[16.4];
2019	862[90.8]	87[9.2];
2020	917[90.3]	97[9.7];
2021	314[87.0]	47[13.0];
2022	349[86.7]	53[13.2];
2023	508[86.1]	89[14.9];

### Detection of PTB and Rifampicin Resistance TB

The overall magnitude of PTB during the study period was 11.7% (434/3696), of which 8.1% (35/434) were RR-TB. A higher magnitude was observed in 2018 (16.4%), followed by 2023 (14.9%) and 2022 (13.2%). The sex-based proportion of PTB cases was similar (male: 11.5%; female: 12.1%). PTB/HIV co-infection was observed in 40.7% of the HIV-positive participants (Table 1).

### Trend of PTB detection and Rifampicin-Resistant TB

**Fig. 1:**
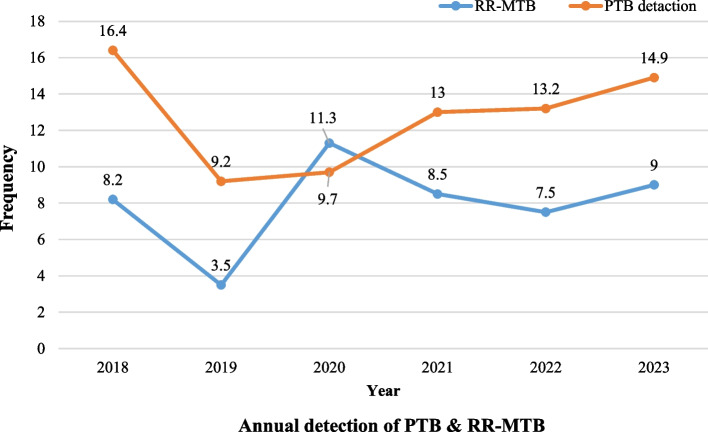
Trend of PTB and RR-MTB in Central Tigray, Ethiopia from 2018 to 2023

PTB a considerable decrement was observed, from 16.4% in 2018 to 9.2% in 2019. However, the trend analysis of PTB showed a statistically significant increase in the prevalence of PTB detection from 9.7% to 14.9% (p = 0.021) in the last four study years (2020–2023). This indicates that more than 30% of PTB cases have been observed in the last four years. Overall, in the past six years observed that there was an increasing rate of PTB with slight fluctuations. Even though it was not statistically significant, the number of RR-TB positive cases indicates an increment from 2020 to 2023 (Fig. 1).

### Distribution of PTB among participates age groups

**Fig. 2:**
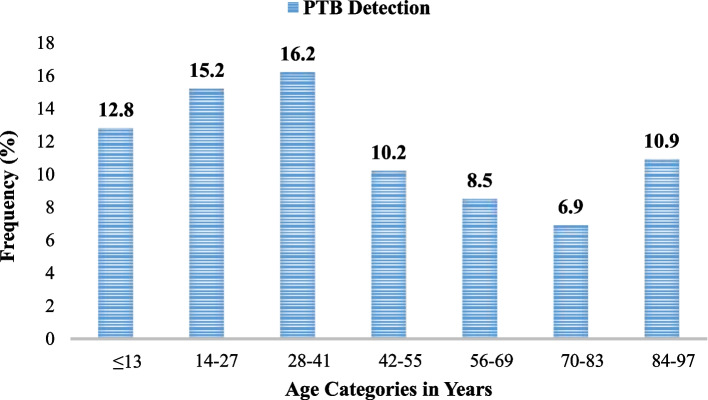
Distribution of PTB among participants age groups in Central Tigray, Ethiopia from 2018 to 2023

During the study period, a high prevalence of PTB was observed among participants age group–28–41 years (16.2%) followed by 14–27 years (15.2%). A comparatively low prevalence of PTB (6.9%) was detected in patients aged 70–83 years (Fig. 2).

### Factors associated with PTB

Binary and multivariable logistic regression models with age of 14–27 years [AOR = 1.90, 95% CI: 1.42–3.43, *p* = 0.001]; 28–41 years [AOR = 2.32, 95% CI: 1.74–4.19, *p* = 0.001], Having HIV [AOR = 10.6, 95% CI: 6.5–17.1, *p* = 0.001], and being presumptive for MDR-TB[AOR = 1.6, 95% CI: 1.1–2.4, *p* = 0.001] were statistically associated with the presence of high PTB prevalence. Participants with urban residences were 51% less likely [AOR = 0.49, 95% CI: 0.39–0.59, *p* = 0.001] to develop TB infection than those with rural residences (Table 2).

**Table 2:** Bivariate and multivariable analysis of factors associated with PTB among presumptive patients in Central Tigray, Ethiopia from 2018 to 2023 (*n* = 3696)

Variables	Status of Tuberculosis		COR [CI, 95%]	*P*-value	AOR [CI, 95%]	*P*-value
	**Negative** **N [%]**	**Positive N [%]**				
**Sex:**						
Male	1949[88.5]	254[11.5]	1.05[0.86–1.29]	0.626		
Female	1313[87.9]	180[12.1]	1			
**Age:**						
≤ 13	136[87.8]	20[12.8]	1			
14–27	429[84.8]	77[15.2]	1.22[1.01–3.09]	0.043	1.90[1.42–3.43]	0.001*
28–41	765[84.4]	148[16.2]	1.28[1.41–3.21]	0.037	2.32[1.74–4.19]	0.001*
42–55	802[89.8]	91[10.2]	0.88[0.51–1.60]	0.824	1.31[.88–1.97]	0.190
56–69	682[91.5]	63[8.5]	0.62[0.53–1.66]	0.791	0.98[0.76–1.65]	0.131
70–83	407[93.1]	30[6.9]	0.47[0.30–0.54]	0.074	0.81[0.66–1.58]	0.19
84–97	41[83.6]	5[10.9]	1.15[1.03–4.2]	0.052	1.6[2.02–6.9]	0.062
**HIV Status:**						
Negative	1570[84.7]	284[15.3]	1			
Positive	51[59.3]	35[40.7]	3.79[2.42–5.94]	0.001	10.6 [6.5–17.1]	0.001*
Unknown	1641[93.5]	115[6.5]	0.39[0.31–0.49]	0.07	0.91[0.63–1.2]	0.24
**Presumptive:**						
PTB	2911[89.7]	336[10.3]	1			
MDR/RR-TB	351[78.2]	98[21.8]	2.4(1.06–4.25	0.001	1.6[1.1–2.4]	0.001*
**Residence:**						
Rural	2052[91.2]	198[8.8]	1			
Urban	1210[83.7]	236[16.3]	2.02[1.65–2.47]	0.001	0.48[0.39–0.59]	0.001*

^*^Statistically significant; 1: reference

## Discussion

Tuberculosis (TB) is a common cause of death around the globe [6]. Most TB cases and deaths occur in developing countries like Ethiopia [7, 8].

In the present study, the overall magnitude of PTB was 11.7%, which is comparable to previously reported results from other parts of Ethiopia: Tigray (9.9%) [11], Eastern Amhara (11%) [15], Central Ethiopia (11.2%) [25], Addis Ababa (13.5%) [26], Mizan Tepi (12.6%) [27], Debre Brhan (13%) [28] and Northwest Ethiopia (14.6%) [29]. However, the current results were lower than previous findings conducted in Tigray (21%) [30], (24.3%) [21], Wolkite town (27.4%) [31], Awi Zone (18.7%) [32], Bahir Dar (21.7%) [33], Gondar (24.6%) [34], Bale (40.4%) [35], Tepi (58.6%) [36], Harar Town (61.2%) [10], Debre Markos (23.2%) [37] Nigeria (22.9%) [38], (27.5%) [39] and China (47.5%) [30]. However, it was higher than studies conducted in Ethiopia: Dessie and Debre Brhan towns (2.6%) [40], Tigray (7.9%) [41] and Gondar (6.3%) [42]. The possible explanation for the variations in the proportions of PTB might be due to differences in methodology (method of diagnosis, study setting, study period, sample size and study participants), environmental conditions, overcrowding, variation in the degree of TB/HIV co-infection and TB management practices. For instance, the high prevalence of PTB in the studies conducted in Bahir Dar (21.7%) [33], Bale (40.4%) [35], Tepi (58.6%) [36] and Harar Town (61.2%) [10] could be because of their methodological technique was microscopy unlike this study (GeneXpert). Another possible reason for the lower prevalence PTB in other studies done in Dessie and Debre Brhan towns [40], Tigray [41] and Gondar (6.3%) [42] could be because of variations in sample size[41], study setting (community based vs hospital based) [40] and methods of diagnosis(culture and microscopy vs GeneXpert) [42] in addition to environmental differences.

RR-TB is a serious health problem and a challenge for TB treatment, especially in low income settings [34]. The present study revealed 8.1% RR-TB, which is less comparable with studies carried out in Ethiopia: Tigray (8.7) [11], (9%) [41], (9.1%) [21], Addis Ababa (9.8%) [26], Eastern Amhara (8.3%) [15], northwest Ethiopia (9.3%) [29], Debre Markos (10.3%) [37] and Nigeria (10.5%) [39]. But our finding is lower than previous reports from Ethiopia: Wolkite Town (15.2%) [31], Gondar (15.8%) [34], Addis Ababa (43.5%) [7]; Nigeria (14.7%) [38]; Congo (42.2%) [43] and China (17.6) [44]. Conversely, our findings were higher than those of studies conducted in Debre Berhan (5.2%) [31], Mizan Tepi (5.7%) [30], Zambia (5.9%) [45] and Kenya (3.6%) [46].The high occurrences of RR-TB in the previous studies of Addis Ababa (43.5%) [7] and China (17.6) [44] could be due to their study subjects were more MDR-TB presumptive unlike to participants of this study who were dominated with presumptive to TB patient. Moreover, greater prevalence of RR-TB in Wolkite (15.2%) [31], Gondar(15.8%) [34], Nigeria (14.7%) [38] and Congo (42.2%) [43] might be due to differences in studies setting (health center vs hospital based), sample size and TB controlling approaches. Another probable reason for the difference in prevalence RR-TB in between this study and other prior studies such as [45] and [46] was the scope of GeneXpert in using for TB diagnosis. Previously GeneXpert assay was recommended only for patients with presumptive MDR-TB. Whereasrecently (including data of this study) GeneXpert MTB/RIF Assay was recommended for every TB presumptive patient.

Consistent with earlier documented findings in Ethiopia [11, 21, 36, 47], our study revealed that PTB frequency decreased from 16.4% in 2018 to 9.2% in 2019. This decrease in the frequency of PTB might be due to improved TB management programs followed by national and regional health bureaus. Contrary to the aforementioned results, the trend analysis of PTB showed a statistically significant increase in the prevalence of PTB detection from 9.7% to 14.9% (*p* = 0.021) in the last four study years (2020–2023). This indicates that more than 30% of PTB cases have been observed in the last four years. A possible explanation for the considerable increase in PTB in the current study could be the destructive civil war that occurred in Tigray, Northern Ethiopia. More than 78% of health facilities in Tigray were not functional [23]. Thus, the war in Tigray had a direct and devastating impact on the delivery of health care services to society. Moreover, it has not only influenced healthcare delivery but also observable economic and psychological trauma to society, which might lead people to be vulnerable to communicable diseases such as TB. Furthermore, the overcrowding of many internally displaced people within small-sized housing as a result of the war could also be another factor for an increasing trend of PTB in the study years [23].

In line with studies conducted in other parts of Tigray [11, 21], Addis Ababa [26], southwestern Ethiopia [36], central Ethiopia [25], Tepi [40], Gondar [34, 42] and Debre Berhan [28], the prevalence of PTB was two-fold higher in the age group of 15–29 (16.6%, *p* = 0.001), followed by 30–44 (15.4%, *p* = 0.001) compared to individuals aged 14 years and below. This could be because people found at this productive age might have a greater chance of being exposed to PTB owing to frequent place-to-place mobility and higher social interaction. Likewise, young individuals are more at risk for HIV due to increased risky sexual behaviors and trigger TB acquisition in youngsters as a co-infectious [33]. Furthermore, this result indicates that TB is an excessive economic burden on societies in developing countries, primarily affecting the productive age groups.

The present study revealed that participants with HIV infection were more likely [AOR = 10.6; 95% CI: 6.5–17.1; p = 0.001] to be infected by PTB than those who were not infected with HIV. In support of our results, studies conducted in other parts of Tigray [11, 41], central Ethiopia [25], Harar Town [10] and Addis Ababa [26] have reported that HIV infection is significantly associated with the occurrence of PTB. This could be because HIV infection boosts the development of TB cases and activation of TBI. Likewise, in this study, being presumptive to MDR-TB was another factor that showed a statistically significant association [AOR = 1.6; 95% CI: 1.1–2.4; *p* = 0.001] with the presence of PTB among the study participants. This finding is in agreement with previous studies conducted in other parts of Ethiopia [11, 37]. This could be due to the acquisition of resistant mycobacteria from the drug resistance TB contact and treatment failure. Moreover, participants with urban residences were approximately 50% less likely [AOR = 0.49; 95% CI: 0.36–0.66; *p* = 0.001] to develop PTB than those who lived in rural areas. Although not statistically significant, an earlier study conducted in Tigray showed that the proportion of PTB was higher among participants with rural residence [20]. A possible reason could be that urban residents might have a relatively better knowledge of TB prevention.

### Limitation of the study

The main limitations of this study was using a retrospective (secondary) data from laboratory TB registration logbooks. Data missing and incompleteness was also another restrictions. Moreover, the factors included for association were limited.Variables like history of TB, participant’s educational status and living condition were not included due to lack of enough information.

## Conclusions

In this study, the overall PTB and RR-TB detection rates were 11.7% and 8.1%, respectively. An increasing trend of PTB was observed in this study, mainly in the recent four years (2020–2023). Though, it was not statistically significant the number of positive observed cases reveals an increment of RR-TB with considerable fluctuations. Age (15–29 and 30–44 years), HIV infection, presumptivity for MDR-TB, and residence were significantly associated with the presence of PTB. Urban residence was associated with a low PTB prevalence. Therefore, we recommend that more attention be given to TB treatment and a continuous monitoring approach should be followed to bend the trends of PTB and RR-TB in the study area.

## Data Availability

All data used and analyzed in this study are available from the corresponding author and can be obtained upon reasonable request.

## Abbreviations

AOR: Adjusted odds ratio
CI: Confidence interval
COR: Crude odds ratio
HIV: Human immunodeficiency virus
SD: Standard deviation
MDR-TB: Multidrug-resistant
MTB: Mycobacterium tuberculosis
SPSS: Statistical Package of Social Science
PTB: Pulmonary tuberculosis
WHO: World Health Organization
